# Mitigating Age-Related Cognitive Decline and Oxidative Status in Rats Treated with Catechin and Polyphenon-60

**DOI:** 10.3390/nu16030368

**Published:** 2024-01-26

**Authors:** Silvia Tejada, Fiorella Sarubbo, Manuel Jiménez-García, Margarida R. Ramis, Margalida Monserrat-Mesquida, Maria Magdalena Quetglas-Llabrés, Xavier Capó, Susana Esteban, Antoni Sureda, David Moranta

**Affiliations:** 1 Laboratory of Neurophysiology, University of the Balearic Islands, 07122 Palma de Mallorca, Spain; silvia.tejada@uib.es (S.T.); mariafiorella.sarubbo@hsll.es (F.S.); manuel.jimenez@uib.es (M.J.-G.); margaramis87@gmail.com (M.R.R.); susana.esteban@uib.es (S.E.); david.moranta@uib.es (D.M.); 2 CIBER Fisiopatología de la Obesidad y Nutrición (CIBEROBN), Instituto de Salud Carlos III (ISCIII), 28029 Madrid, Spain; 3 Health Research Institute of Balearic Islands (IdISBa), 07120 Palma de Mallorca, Spain; margalida.monserrat@uib.es (M.M.-M.); m.quetglas@uib.es (M.M.Q.-L.); xaviercapofiol@hotmail.com (X.C.); 4 Research Group in Community Nutrition and Oxidative Stress (NUCOX), University of Balearic Islands, 07122 Palma de Mallorca, Spain

**Keywords:** antioxidant enzymes, Barnes maze test, cognitive decline, lipidic damage, polyphenols

## Abstract

Aging is a normal physiological process influenced by the combination of multiple mechanisms, primarily oxidative stress and neuroinflammation, which impact general physiology and brain function. Phenolic compounds have demonstrated the ability to slow down the aging process of the brain due to their antioxidant and anti-inflammatory effects. This study assessed the protective properties of catechin and polyphenon-60 in non-pathologically aged rats regarding visuo-spatial learning and the oxidative status of the frontal cortex. Old animals were treated with catechin or green tea extract (polyphenon-60) for 36 days, daily. Healthy old and young rats were used as controls. During the first training phase, treated rats executed the test better, locating the target in less time compared with the controls. Biomarkers of oxidative stress (catalase activities, superoxide dismutase, glutathione reductase, and glutathione S-transferase) were reduced in the brain of old animals, although their activities were partially improved after both antioxidant treatments. Furthermore, the rise in the production of reactive oxygen species and malondialdehyde levels—a marker of lipid peroxidation—in the frontal cortex of aged animals was significantly ameliorated after the interventions. In conclusion, old rats exhibited enhanced cognitive function and reduced stress levels following the administration of catechin and polyphenon-60.

## 1. Introduction

Population aging is increasing worldwide, with an estimated 11% of the world population being over 60 years old, and this could increase up to 22% by 2050 [1,2]. Since the year 2000, the percentage of the population aged 65 or over has increased in all regions of the world, and in some of them, it will even double by 2030 (e.g., from 6% to 12% in Asia) [2]. Aging is a normal physiological process, but it is also accompanied by a decrease in motor, cognitive, or sensory skills [3,4], as well as an increase in different pathologies, such as cardiovascular and metabolic diseases [5]. Consequently, people’s quality of life of decreases, and morbidity increases. The brain is an especially sensitive tissue that can be severely and frequently affected by aging [6]. At the cellular level, events such as abnormal myelination, astrogliosis, or oligodendrogliosis, among others, promote a state of chronic inflammation directly associated with increased microglial activation. In turn, a decrease in processes such as synaptic plasticity, neurogenesis, or the number of neurotransmitters has also been observed [7]. Therefore, changes related to cognitive impairment and neurodegeneration occur, including DNA damage, lysosomal dysfunction, loss of protein homeostasis, or immune dysregulation [8]. Aging is also influenced by oxidative stress mechanisms originating in the cells. Taking into account that free radicals increase during old age, they are considered to be a pivotal part of the aging process [9]. The increase in oxidative species can promote lipid and DNA damage and deficits in protein metabolism [9]. To counteract the increase in reactive oxygen species (ROS), the organisms have several enzymatic antioxidant mechanisms such as catalase (CAT), superoxide dismutase (SOD), glutathione peroxidase (GPX), or glutathione reductase (GR), and non-enzymatic mechanisms such as glutathione (GSH) [10]. SOD decomposes the superoxide anion into hydrogen peroxide, which is further metabolized to molecular oxygen and water by GPX and CAT, protecting against an excess of reactive species [11]. Nevertheless, when an excessive ROS production occurs, the antioxidant mechanisms could be overwhelmed, causing oxidative stress [12].

Dietary interventions have been described to decelerate the brain’s aging process [13]. Therefore, an approach to stimulate healthy brain aging would be changes towards the consumption of healthy foods. Natural products have been gaining interest in recent years, mainly those of herbal origin, since they fulfil functions related to disease prevention and health promotion [13,14,15]. Among them, polyphenols have been described as a promising tool to mitigate and counteract the processes related to aging and its associated diseases [14,15,16]. Polyphenols are chemical substances found in fruits, vegetables, and some drinks such as coffee, wine, or tea [17]. All around the world, one of the most consumed beverages is green tea, which is characterized by the presence of a wide range of polyphenolic components, mainly a group called catechins [18]; however, there are more relevant sources of catechins such as berries, cocoa [19], or pomegranates [20]. In addition, there are commercial extracts of green tea, such as polyphenon-60, which are composed of the main active polyphenolic compounds [21]. The ways in which polyphenols are capable of acting as immunomodulatory agents has been described, including triggering intracellular signaling pathways such as the nuclear factor kappa-B (NF-κB), the activation of protein kinases by mitogens (MAPK), and several other processes involved in epigenetic modulation [22]. Polyphenols also have a neuroprotective ability as they are involved in reducing inflammation and oxidative stress [23,24]. In turn, these compounds help improve cognitive processes such as memory [25]. The use of catechins has shown antioxidant effects both in cardiovascular and neurodegenerative diseases, and also in carcinogenic events, acting directly (removing ROS or chelating metal ions) or by indirect mechanisms, inducing the production of antioxidant enzymes or inhibiting prooxidants [26,27]. However, in relation to catechin and polyphenol-60, information is lacking related to their beneficial effects on non-pathological aging, which has been found, as in other polyphenol compounds, to be an important way of assessing their potential as antiaging molecules. As such, the aim of the current study was to evaluate the brain-protecting effects of catechin and polyphenon-60 in a healthy animal model by studying visuo-spatial learning together with the oxidative status of the brain.

## 2. Materials and Methods

### 2.1. Animals and Treatments

Twenty-four male Wistar rats were housed individually and maintained under standard conditions with a 12:12 (L:D) schedule, having free access to food and water, at a temperature of 20 ± 2 °C and 70% humidity. All measures were taken to lessen the suffering of animals and decrease the number of animals used. All procedures were performed following the European Convention for the Protection of Vertebrate Animals used for Experimental and other Scientific Purposes (Directive 86/609/EEC) and according to the guidelines of the Bioethical Committee of the University of the Balearic Islands (2014/05/AEXP).

Animals were randomly divided into four groups (n = 6) based on age. All treatments were intraperitoneally administrated. Two controls groups were used, young rats (three months old) and old control rats (20 months old). Two groups of old rats (20 months old) were used as the experimental groups. The control groups received corn oil (1 mL/kg i.p.). A pilot study evaluating the administration of the corn oil (dose, administration route, and time) was performed previously, observing no alterations in behavioral or neurochemical parameters, and these results were presented at the *XXXVII Congress of the Sociedad Española de Ciencias Fisiológicas* (SECF, Granada, Spain, 2014) and the 9th FENS Forum of Neuroscience (Milan, Italy, 2014) [28]. For 36 days, the experimental old rats were daily treated with (+)-catechin (2R,3S)-2-(3,4-dihydroxyphenyl)-3,4-dihydro-1(2H)-benzopyran-3,5,7-triol (20 mg/kg i.p.; Sigma-Aldrich, Darmstadt, Germany) or green tea extract (polyphenon-60, 40 mg/kg i.p.; Sigma-Aldrich). According to the supplier’s declaration (Sigma-Aldrich), polyphenon-60 contains a minimum of 60% of total catechins (≥60%). The content of catechin derivatives in polyphenon-60 extracted from green tea is as follows: epigallocatechin gallate (EGCG) 28.8%, epigallocatechin (EGC) 19%, epicatechin gallate (ECG) 7%, epicatechin (EC) 6.4%, gallocatechin (GC) 5.2%, gallocatechin gallate (GCG) 2.1%, catechin (C) 1.4%, catechin gallate (CG) 0.3% [21]. The polyphenon-60 dose was selected following the methodology described in a previous work by Ramis and collaborators [29], being in the range of previous works without toxic effects [30,31].

There were no animal exclusions and all of them finished the experiment. Rats were sacrificed by decapitation one day after the performance of the last cognitive test. The brains were quickly removed, and the frontal cortex was separated and maintained at −80 °C until analysis.

### 2.2. Barnes Maze Test

The behavioral test was conducted between 9:00 and 12:00 h. The analyses were carried out on the rats individually; and to prevent potential masked effects related to chronobiology, animals from various experimental groups were alternated. The visuo-spatial memory was evaluated using the Barnes maze test. This cognitive test consists of a circular elevated board 130 cm in diameter, with 18 holes uniformly distributed along the outer border. One of the holes has a box below it, not visible from the center of the board, so the rat should move closer or put its head inside to recognize it. This box is considered the target box. The goal of this test is to evaluate the capability of rats to learn where the target is placed, using their visuo-spatial memory. Spatial reference points are provided in the room to facilitate the search for the target. To stimulate the search for the target, an aversive stimulus is used, in this case a non-harmful powerful light which increases the agoraphobia of the rats [32]. This light is on every time that the animal explores the platform and is off when it reaches the target. Following previous studies [33,34,35], Barnes maze comprises a total of three phases: the habituation, the training, and the test phase. In all phases, the animal is positioned in the midpoint of the maze, the light turns on, and the researcher observes how the animal finds the target. The habituation phase takes place one day before the training and the test. In this phase, the animals are habituated to the maze, letting them explore it until they find the target or until a maximum of 3 min. If the target is found, the animal is allowed to remain in the box for 1 min; if not, it is manually directed into the box, in which it also remains for 1 min. The habituation phase has the objective of reducing the anxiety usually generated by new spaces in rodents. Two consecutive phases are performed 24 h after the habituation; first, the training, the aim of which is to let the animal learn the position of the target; and the final test phase, the aim of which is to corroborate if the animal has learned the position of the target. In the training phase, each rat performs 3 trials, separated by 10 min. Each trial ends after the rat enters the target or after 3 min if the rat does not find it. Finally, the test phase is performed 10 min after the last training trial; and lasts until the rat finds the target or for a maximum of 60 s if the target is not found. The test was conducted at the end of the treatments, with the habituation phase occurring on the 35th day and the test phase taking place on the last day.

The parameters registered during training and test (measured as indicators of learning) were as follows: latency, number of errors, and strategy to find the target. Latency was defined as the time the rats spent to find the target, measured in seconds. Errors were referred to as the total number of holes explored that were not the target. Both latency and errors were compared with young control animals to assess the effect of aging and treatments. In addition, the strategy used to find the target was considered as mixed (without following an order, it was random), serial (following a pattern), or direct (directly to the target) [36].

Between trials and phases, the board was cleaned with 90% ethanol in order to avoid possible olfactory or solid traces.

### 2.3. Antioxidant Enzyme Activities

The frontal cortex samples were homogenized in Tris-HCl 10 mM (pH 7.5) using a sample dispersing system (Ultra-Turrax^®^ T10 Disperser, IKA, Staufen, Germany) and centrifuged at 9000 rpm for 10 min at 4 °C. Supernatants were collected and used for all the biochemical analyses. The activities of catalase (CAT), superoxide dismutase (SOD), glutathione reductase (GRd), and glutathione peroxidase (GPx) in the frontal cortex were evaluated using a Shimadzu UV-2100 spectrophotometer (Shimadzu Corporation, Kyoto, Japan) at 37 °C. The spectrophotometric method of Aebi [37] based on the decomposition of H_2_O_2_ was used to evaluate the CAT activity. An adaptation of the method of McCord and Fridovich [38] was used to measure the SOD activity. A modification of the Goldberg and Spooner [39] spectrophotometric method was used to measure the GRd activity. An adaptation of the spectrophotometric method of Flohe and Gunzler [40] was used to measure the GPx activity. Protein concentration was determined using a commercial kit (Merk Life Science S.L.U., Madrid, Spain) which was used to normalize the activity values.

### 2.4. ROS Production

ROS production was evaluated in the brain homogenates by means of 2,7-dichlorofluorescindiacetate (DCFH-DA) as an indicator in a 96-well microplate [41]. The fluorescence (excitation 480 nm; emission 530 nm) was monitored for 1 h at 37 °C in FL9800 Microplate Fluorescence Reader (Biotek Instruments, Inc., Madrid, Spain).

### 2.5. Malondialdehyde Determination

Malondialdehyde (MDA), as indicator of lipid peroxidation, was determined in the frontal cortexes of the rats through a colorimetric analysis relaying on the reaction of MDA with a chromogenic reagent to produce a stable chromophore with maximal absorbance at 586 nm. In brief, standards or samples were introduced to glass tubes containing n-methyl-2-phenylindole (10.3 mM) in acetonitrile:methanol (3:1). HCl 12 N was then introduced, and the samples underwent incubation for 1 h at 45 °C. The absorbance at 586 nm was assessed using an Epoch microplate spectrophotometer (Bio-Tek, Agilent Technologies, Madrid, Spain).

### 2.6. Glutathione Redox Status

Total glutathione (tGSH) and glutathione disulphide (GSSG) levels were assessed kinetically in frontal cortex samples at 412 nm and 37 °C in a spectrophotometer (UV-2100, Shimadzu Corporation, Kyoto, Japan) following a previously described method [42]. Briefly, tGSH levels were determined in deprotreinized samples to monitor lessening DNTB in the occurrence of NADPH and glutathione reductase. GSSG analysis was assessed adding vinil piridine to the samples and following the same procedure described above. GSH content was determined by subtracting GSSG (in GSH equivalents) from the tGSH concentration.

### 2.7. Western Blot Analysis

Manganese superoxide dismutase (MnSOD) protein levels in brain tissue were determined by Western blot. Twenty microgram protein aliquots were separated on a 15% acrylamide sodium dodecyl sulfate (SDS) polyacrylamide gel and subjected to electrophoresis at 200 V for 90 min. Using a Trans-Blot^®^ Turbo™ Transfer System (Bio-Rad, Madrid, Spain) bands were electrotransferred onto a nitrocellulose membrane. The membrane was then blocked (5% non-fat powdered milk in PBS, pH 7.5) for 5 h and incubated with the primary antibodies for MnSOD (1:5000, Calbiochem San Diego, CA, USA) and β-actin (1:200; Santa Cruz, CA, USA) as the loading control. Following primary antibody incubation, the membrane was incubated with fluorescent secondary antibodies IRDye 800CW (1:5000; LI-COR Biosciences, GmbH, Bad Homburg vor der Höhe, Germany). Images acquisition was performed using the Odyssey infrared imaging system (LI-COR Biosciences, GmbH, Germany) and analysis was carried out using the public domain software ImageJ 1.53e. A molecular weight marker, Precision Plus Protein Kaleidoscope™ (Bio-Rad, Madrid, Spain), was used.

### 2.8. Statistical Analysis

SPSS^®^ (v.25 for Windows^®^) was used for statistical analysis using two-way ANOVA for statistical evaluation of behavioral results using Bonferroni post hoc tests for pairwise statistical comparisons, and using one-way analysis of variance (ANOVA) for biochemical analysis. For the continuous variables, it was followed by the Bonferroni post hoc test, and for categorical variables the Chi square was applied. Values are expressed as mean ± SEM and percentage. A result considered statistically significant a *p* < 0.05.

## 3. Results

### 3.1. Barnes Maze Test

**Figure 1:**
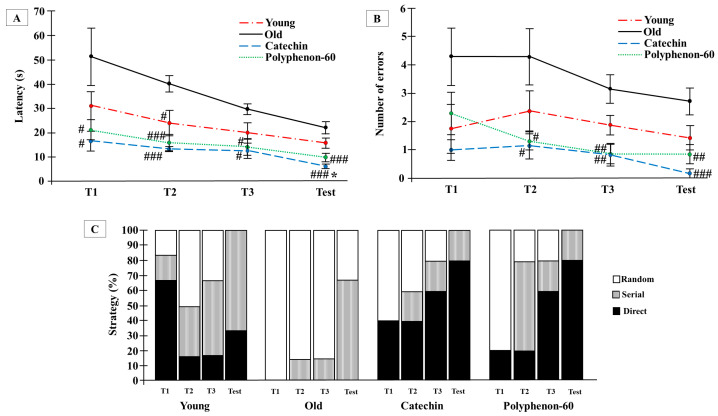
Effects on visuo-spatial learning ability of rats evaluated by means of the Barnes maze test. (**A**) Latency: time spend to finish the test; (**B**) number of errors committed during the test; (**C**) strategy used to find the target in percentage. Latency and number of errors are represented as mean ± SEM and strategy by %. Two-way analysis of variance (ANOVA) and chi-square test. * *p* < 0.05 with respect to young control; and # *p* < 0.05; ## *p* < 0.01; ### *p* < 0.001 with respect to old control. T: Training (performed the same day of the test).

The Barnes maze test was used to analyze visuo-spatial learning after treatments with catechin and polyphenon-60. The test was only performed at the end of the treatments in order to avoid any possible learning produced by the successive training that the test execution can imply. When latency was assessed with two-way ANOVA, significant effects for the treatment (F(3,90) = 20.508, *p* < 0.0001) and the training/test (F(3,90) = 8.735, *p* < 0.0001) were detected, but not for the interaction (F(9,90) = 0.777, *p* = 0.638). The latency to complete the test (Figure 1A) indicated that the animals treated with both catechin and polyphenon-60 performed the test more effectively from the first training phase (*p* < 0.05), finding the target in less time compared with old control rats. For the old, treated rats, the time taken to find the hole when performing the test was statistically reduced (*p* < 0.01 for polyphenon-60 group, *p* < 0.001 for catechin group) with respect to the old control group. In addition, learning was observed in all groups between the first training phase and the test, showing statistical differences with the first training phase for the old groups (*p* < 0.05). Differences between old, treated rats and the young group were only observed in the latency during the test phase for the catechin treated group (*p* < 0.05). The committed errors showed a similar pattern to that for latency with significant effects of the treatment (F(3,95) = 15.523, *p* < 0.0001) and the training/test (F(3,95) = 2.819, *p* < 0.043) but not for the interaction (F(9,95) = 0.048, *p* = 0.928) (Figure 1B). Significant differences for errors were found between the second training phase and the test phase in the old animals treated with catechin (*p* < 0.05 for training 2, *p* < 0.01 for training 3, *p* < 0.001 for test) and polyphenon-60 (*p* < 0.05 for training 2, *p* < 0.01 for training 3 and test) compared to the old control group. Attending to the number of errors from the first training to the test phase, no differences were observed in the young and the old control groups; however, the errors committed by the catechin group were diminished during the test compared to the second training phase (*p* < 0.05); the old animals that received polyphenon-60 also had a reduced number of errors, which was significant between training phases 1 and 3 (*p* < 0.05). Regarding the strategies used to complete the task, the old control rats used a random strategy to locate the target during the first training phase (100%), and random and serial ones in the following trainings and tests. In contrast, the old, treated animals used different strategies to find the escape box during the trainings, and used serial and direct approaches during the test. During the first training phase, the direct strategy was followed by 40% of the animals treated with catechin, and by 20% of those treated with polyphenon-60; and during the test, 80% of the animals in both catechin and polyphenon-60 groups used the direct strategy, while none of the animals in the old control group used this strategy (*p* < 0.05) (Figure 1C).

### 3.2. Oxidative Status

Regarding oxidative status (Table 1), the results showed the highest levels of ROS in the frontal cortexes of untreated old rats compared to the young rats (*p* < 0.001), and these levels were also higher after the catechin (*p* < 0.01) and polyphenon-60 (*p* < 0.05) treatments, with lesser differences compared to the young group. In addition, MDA levels were increased in the frontal cortexes of the old animals and this lipid damage was partially reversed after the treatments, especially with polyphenon-60.

**Figure 2:**
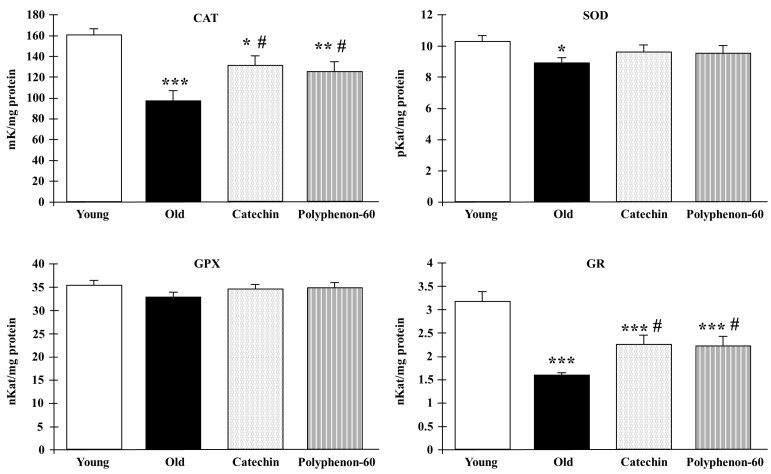
Antioxidant enzyme response in frontal cortexes of young control, old control, and treated rats (catechin and polyphenon-60) (3 and 20 months old, respectively). Data is expressed as mean ± SEM. * *p* < 0.05; ** *p* < 0.01; *** *p* < 0.001 respect to young control; and # *p* < 0.05 respect to old control rats. One-way analysis of variance (ANOVA) and Bonferroni post hoc test. CAT: catalase; SOD: superoxide dismutase; GPX: glutathione S-transferase; GR: glutathione reductase.

The activities of the biomarkers of oxidative stress CAT, SOD, GR, and GPX in the frontal cortexes of the rats are shown in Figure 2. The results for the old control rats revealed a statistically significant reduction in the activity of CAT (*p* < 0.001), SOD (*p* < 0.05), and GR (*p* < 0.001) in comparison to the young rats. However, the treatment of the old animals with catechin and polyphenon-60 increased the enzymatic activities compared to the untreated aged rats, being closer to the values of the young rats, although differences were also found between treated rats and the young group in CAT (*p* < 0.05) and GR (*p* < 0.001) activities. No differences were found in the GPX activity.

**Figure 3:**
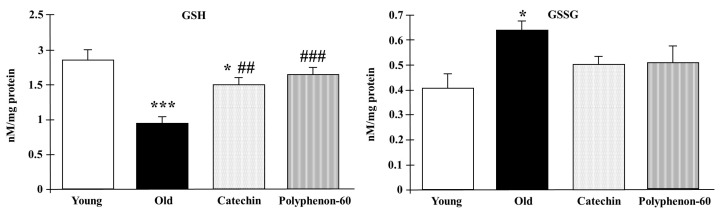
GSH and GSSG levels in frontal cortexes of young control, old control, and treated rats (catechin and polyphenon-60) (3 and 20 months old, respectively). Bars represent mean ± SEM. * *p* < 0.05 and *** *p* < 0.001 respect to young control; and ## *p* < 0.01 and ### *p* < 0.001 respect to old control rats. One-way analysis of variance (ANOVA) and Bonferroni post hoc test.

GSH and GSSG levels in the frontal cortexes of the rats are shown in Figure 3. Old control rats presented lower levels of GSH compared to the young control group (*p* < 0.001). However, old rats showed an increase in the GSH levels after both catechin and polyphenon-60 treatments compared to the untreated old rats (*p* < 0.01 and *p* < 0.001, respectively) with values closer to the younger rats. With respect to GSSG, the old control rats showed higher levels compared the young control group (*p* < 0.001). Moreover, the old, treated rats seemed to show a tendency towards an increase in these levels compared to the young rats, but it was not statistically significant.

**Figure 4:**
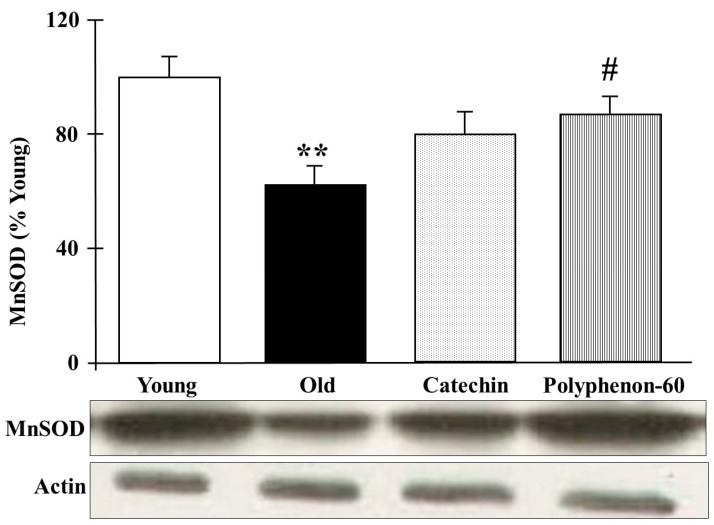
Western blot analysis of cortical levels of MnSOD young control, aged control, and treated rats (catechin and polyphenon-60) (3 and 20 months old, respectively). Data is represented as mean ± SEM of protein levels expressed as percentage relative to the young control group. ** *p* < 0.01 compared to the young control; and # *p* < 0.05 with respect to old control rats. One-way analysis of variance (ANOVA) and Bonferroni post hoc test.

The protein levels of mitochondrial MnSOD in the frontal cortexes of the rats are represented in Figure 4. Old control rats presented with significantly lower protein levels compared to the young group (*p* < 0.01). Nevertheless, the old rats showed an increase in the MnSOD levels after both catechin and polyphenon-60 treatments compared to the old control group, although the changes were only statistically significant for polyphenon-60 (*p* < 0.05).

## 4. Discussion

Physiological and cognitive decline related to aging can impact life expectancy and quality, leading to deficits in attention, working and episodic memory, or spatial learning, even in non-pathological aging [1,3]. Given the growing aging population, there is a need for new strategies to address the associated social and health costs. Polyphenols are bioactive compounds with antioxidant and anti-inflammatory properties [43,44]. Green tea, a beverage which is widely consumed globally, possesses health-promoting qualities, hinting to its therapeutic potential in age-related diseases [45,46,47]. Serving as a significant source of polyphenols, green tea is particularly rich in catechins. These catechins can exert neuroprotective effects due to their ability to cross the blood–brain barrier [48,49]. Moreover, polyphenon-60 is an extract from green tea composed of the main active polyphenols in green tea [21]. In the current work, the learning and antioxidant system deficits related to aging improved after the treatments with catechin and polyphenon-60 in old rats.

The obtained results showed a positive effect of the catechin and polyphenon-60 treatments on brain cognitive function. Animals exhibited better performance in the Barnes maze test, indicating improved visuo-spatial learning. This task requires the integration of both sensitive and motor information, for which the frontal cortex is involved; in fact, normal aging in rats is associated with impaired medial frontal cortex function [50]. The observed differences in the latency and number of errors committed in the test, together with the use of a more direct strategy (from the first training of the test phase), indicated that the treated old animals remembered the location of the target better than the old control group. A previous work, using the same treatment protocol and age of rats, showed a beneficial cognitive effect when animals were treated with these compounds. Concretely, a better visuo-spatial working memory was observed in old rats after thirty-six days of catechin and polyphenon-60 treatments, as evidenced by the radial maze test that was performed in less time with fewer errors compared to untreated old rats. Additionally, the episodic memory studied by the novel object recognition test showed a higher discrimination between the novel and familiar objects at the end of the treatments [29]. In another work, old rats (20-month-old) that were fed an antioxidant-enriched diet for 20 weeks demonstrated improved performance in the spatial learning test compared to controls (3 months old). They made fewer errors, employed more-effective searching strategies, and showed enhanced performance in the radial maze test [51]. Altogether, cognitive tests conducted on rodents fed or treated with a high polyphenolic content, as in the current work, exhibited their favorable effect on brain function. Other works in rodent models reported similar results with different cognitive tests. In a previous study [52], old rats (24 months) increased the escape latency and diminished the time spent in the escape quadrant of a water maze test in comparison with young animals, indicating a disruption of learning and memory, but this was significantly reversed after resveratrol administration (50 mg/kg/day orally, 12 weeks). The study also observed an improvement in TNF-α and interleuquine-1b levels, which have been related to dementia in aged individuals [53]. In fact, the anti-inflammatory properties of polyphenolic compounds have been related to multiple pathways that, in turn, are involved in neurogenesis, synaptic plasticity and neurotransmitter signaling [54]. For instance, the consumption of *Aronia melanocarpa* berries juice (10 mL/kg in drinking water), rich in polyphenolic components (mainly antrocyanins, chlorogenic acid, and neochlorogenic), by old rats (24-month-old) improved the memory retention measured by the active avoidance conditioning test during the learning sessions, although only a tendency was observed during the final test; also, increased activity of acetylcholinesterase in the hippocampus of treated rats was related to higher cholinergic nerve fiber density, thus positively affecting the memory consolidation processes [55]. Old mice (18-month-old) fed with a diet containing high amounts of cocoa, nuts, vegetable oils, and flours exhibited improvements in the decreased exploratory activity related to aging which became similar to that of the young animals. Although these old animals showed impaired spatial learning, as evidenced by a decrease in swimming speed and an increase in escape latency in the Morris water maze; the introduction of the diet led to improvement. Overall, these findings suggest a significant cognitive improvement [56]. These results in animal models agree with human evidence, since the consumption of several types of tea by aged people for a long tine showed reduced cognitive impairment, better performance in cognitive tests, and a lower incidence of Parkinson’s disease [57,58,59].

The aging process is characterized by senescence and cellular dysfunction favoring the excessive production of reactive species that can impair different cellular components [60]. The obtained results showed an increase in the ROS production alongside a general reduction in antioxidant defenses in old control rats compared to young ones. These findings, together with the cognitive results, could suggest a positive effect in the cognition of the animals. It is well known that mitochondria are important players in the aging process and most age-related diseases, mainly neurodegenerative ones [61,62]. They are the main organelle that produces ROS and are also the main target of ROS [63]. In accordance with the present results, an increase in ROS production was reported in old rats, mostly derived from mitochondrial complex I dysfunction [64,65,66]. The participation of the mitochondria in the dysfunction associated with aging was evidenced by the decrease in MnSOD levels respect to young animals, indicating a decrease in the ability to eliminate superoxide anions. Similarly, a reduction in MnSOD gene expression and protein levels were also observed in the frontal lobes of 24-month-old rats [64]. Furthermore, the analysis of protein levels shows the existence of an effect on gene expression, and not that the changes in enzymatic activities are solely due to post-translational modulations. In addition to this increase in ROS production, several studies have also shown progressive decreases in enzymatic antioxidants (CAT, SOD, GPX) and GSH, and an increase in GSSG levels in the brain of aged rats, that were reversed after the treatment with ellagic acid or safranal [67,68], similarly to the current work. The evidence suggests an age-related decline in the capability to respond to oxidative stress mediated by the nuclear factor erythroid 2-related factor 2 (Nrf2) pathway and the expression of its downstream antioxidant genes, which could partly explain the decrease observed in aged animals [69]. Taken together, they are also related to increased MDA levels, a biomarker of lipid peroxidation, in old animals; in the current work, this lipid damage was also observed in all aged groups but was partially reversed by the treatments. The rise of the lipid oxidation induces membrane dysfunction due to the high concentration of polyunsaturated fatty acids that are very susceptible to oxidation [70,71]. Consequently, it increases the susceptibility to neuronal damage and functional deterioration with aging [70].

The observed rise in the antioxidant enzymes activity and GSH levels after the treatments were performed may contribute to a reduction in the adverse effects of the ROS increase associated with age. The antioxidant effects of green tea have been described in several works. However, there is not excessive information related to oxidative stress in old rats and only few works relate physiological and biochemical parameters. The intake of an infusion of green tea by old rats (12 and 19 months) showed increased SOD activity compared to the elderly control group [72]. Young rats (5 weeks old) that were treated with scopolamine performed worse in the behavioral and cognitive tests (short-term spatial working, long-term avoidance memories, and long-term spatial learning and memory) than the rats treated with the same drug and epigallocatechin gallate, the main component in green tea. It was accompanied by reduced MDA levels and increased SOD activity in the hippocampus of the rats treated with epigallocatechin gallate [73]. In another study, young adult rats (7–8 weeks old) were used to assess the effects of virgin coconut oil, which is rich in active polyphenol compounds. Spatial learning and memory were significantly improved in the treated rats compared with the controls, and CAT, SOD, and GPX activities, as well as GSH levels, increased in the whole brain while the MDA levels were reduced [74]. Other works were mainly focused on diseases such as Alzheimer’s Disease or high-fat diets [75,76]. For instance, oxidative damage to the synapse in the rat cerebral cortex during aging could play a role in the impairment of cognitive functions. In this sense, antioxidant Vitamin E supplementation in rats improved learning functions (water maze and eight-arm radial maze) prior to the induced stress and avoided a deficit in memory [77]. Also, α-tocopherol improved memory measured by novel-object recognition and conditioned-fear tests, and reduced lipid damage in transgenic mice [78]. The flavonoid naringenin (12 weeks) was used in 10-month-old mice with cognition deficits induced by a high-fat diet. The authors reported better spatial learning and memory evaluated by the Barnes and water maze tests, and a reduction in the oxidative stress measured through reduced lipid damage and increased SOD and GSH activities [76]. Some studies have shown that polyphenols present in tea, such as (−)-epigallocatechin-3-gallate, induced an increase in the endogenous antioxidants in the microglia and neurons through the activation of the antioxidant regulatory elements (ARE)/Nrf2 pathway [79,80]. Moreover, GSH has been shown to be an essential antioxidant to maintain the redox state in the brain, and reduced levels were related to oxidative stress in neurological disorders [81,82]. In another study, prolonged consumption of a green tea infusion (50 mg/kg, 8 months; including (−)-epigallocatechin-3-gallate, (−)-epicatechin, (−)-epicatechin-3-gallate (+)-gallocatechin-3-gallate) also resulted in increased antioxidant enzymes and GSH levels, and a GSSG reduction in the hippocampus of rats compared to the control group. The authors concluded that the effects of the extract would be mediated, in part, by an increase in the activation of cyclic AMP response element-binding, which controls the induction of antioxidant genes and key pro-survival proteins such as brain-derived neurotrophic factor and B-cell lymphoma-2, among others [72]. The increase in GSH levels in parallel to the decrease in GSSG levels after the polyphenol treatment could be not only the result of the recovered GR but also because of the activation of GSH synthesis induced by the Nrf2 pathway [83]. Moreover, the decreased levels of MnSOD in old animals were also recovered after the administration of polyphenolic compounds, which could be related to an improvement in mitochondrial function. An increase in brain MnSOD was also observed in D-galactose-induced oxidative aging in mice after the consumption of a polyphenolic extract of Kuding tea (catechin, caffeic acid, GCG, ferulic acid, isochlorogenic acids B and A) [84]. Additionally, polyphenol administration effectively restrained the progression of pathological aging, as was evidenced by the decline in the ROS production and lipid damage levels. Similarly, chronic administration of catechins from green tea (0.05% in drinking water, 6 months, 80 mg/kg/day) to 14-month-old C57BL/6J mice prevented the reduction in antioxidant enzymes (SOD and GPX) in serum and the oxidative damage in the hippocampus; this treatment also reduced the activation of NFκB [85]. These results are in line with the partial recovery of the antioxidants systems after polyphenolic treatments in aged rats observed in the current work. In contrast, white tea extracts (4 and 12 mg/Kg, 20-month-old rats; caffeine, gallic acid, epigallocatechin-3-gallate, epigallocatechin) did not show significant changes in the degree of carbonylated proteins and hydroperoxides compared to the control group. However, a decrease in these levels was observed compared to the animals with a high oxidative stress induced by an acute administration of adriamycin, which is used to treat different types of cancer [86]. The differences between these studies could be mainly due to the difference between the doses.

The previous studies are in line with the current results, although the age of the rats and the studied region were different. Combining cognitive tests with the evaluation of the oxidative status in an aged rat model, without dietary intervention or with an existent pathology, allows an attempt to study the normal aging process and how catechin and polyphenon-60 could improve the normal aging process. A previous work in our laboratory with the same methodology showed similar results in other cognitive tests and recovering the catecholaminergic and serotonergic systems and some proteins related to memory in hippocampus [29]. However, the frontal cortex is involved in the integration of all the sensitive and motor information, which is necessary for the development of learned tasks, so is important for cognitive flexibility and memory consolidation [87,88]. Altogether, it could be interpreted that the mentioned compounds provided protection and had beneficial effects on brain function by reducing the oxidative status through increasing the antioxidant agents and, in turn, reducing the lipid damage, as has been observed in the frontal cortexes of the non-pathological old rats used in the present work.

## 5. Conclusions

The administration of catechin and polyphenon-60 to aged rats was able to partially reverse age-associated cognitive decline, improving visuo-spatial learning and reducing the pro-oxidative state in the frontal cortex of the old animals. These results are positive indicators of the neuroprotective effect of these compounds, found as principal polyphenolic components in green tea, that may be related to a better neuroplasticity and an oxidative status in elderly people. These results suggest that the oxidative status in non-pathological aged rats could have a central role in the aging-associated cognition impairment, since antioxidant systems were recovered together with an improvement in learning after the treatments. Even though there are studies that have shown that prolonged consumption of beverages rich in polyphenols reduces the risk of cognitive impairment, the underlying mechanisms also require further study. Future research should focus on determining the molecular mechanisms of action of polyphenols and the necessary doses together with different combinations of different bioactive compounds to observe these beneficial effects in humans.

## Figures and Tables

**Table 1:** Levels of reactive oxygen species (ROS) and malondialdehyde (MDA) in frontal cortexes of young, old control, and treated rats (catechin and polyphenon-60) (3 and 20 months old, respectively).

	Young	Old	Catechin	Polyphenon-60
ROS (RFU/(min·mg protein))	2169.9 ± 296.2	4523.6 ± 373.9 ***	3796.9 ± 429.4 **	3705.5 ± 471.8 *
MDA (nM/mg protein)	10.3 ± 1.4	19.6 ± 1.3 ***	14.9 ± 1.6 *,#	14.0 ± 1.5 #

One-way analysis of variance (ANOVA) and Bonferroni post hoc test. Data are expressed as mean ± SEM. * *p* < 0.05; ** *p* < 0.01; *** *p* < 0.001 with respect to young group; and # *p* < 0.05 with respect to old control rats.

## Data Availability

Researchers wishing to access the data used in this study can make a request to the corresponding author: antoni.sureda@uib.es. The data are not publicly available due to maintain the integrity of ongoing research efforts.
